# Application of QBA to Assess the Emotional State of Horses during the Loading Phase of Transport

**DOI:** 10.3390/ani12243588

**Published:** 2022-12-19

**Authors:** Francesca Dai, Maria Giorgia Riva, Emanuela Dalla Costa, Riccardo Pascuzzo, Alana Chapman, Michela Minero

**Affiliations:** 1 Equine Independent Researcher, 20010 Milan, Italy; 2 Dipartimento di Medicina Veterinaria e Scienze Animali (DIVAS), Università degli Studi di Milano, Via dell’Università 6, 26900 Lodi, Italy; 3 Fondazione IRCCS Istituto Neurologico Carlo Besta, Via Celoria 11, 20133 Milan, Italy; 4 World Horse Welfare, Anne Colvin House, Snetterton, Norwich NR16 2LR, UK

**Keywords:** horse, QBA, transport

## Abstract

**Simple Summary:**

The traditional approach to animal welfare has been primarily based on assessing negative factors. However, over the past few years, due to changes in societal beliefs and values and improved scientific understanding of animals, researchers are being asked to develop tools to identify positive animal-based measures and, more in general, to evaluate animals’ emotional states. Transportation, mainly the loading phase, is a crucial welfare concern in horses and the development, enhancement, and implementation of positive animal-based indicators during transport are of critical importance. This study aimed at investigating the accuracy of Qualitative Behaviour Assessment (QBA), using both free choice profiling and fixed list methodologies, in horses during the loading phase of transport. A total of 13 stakeholders were asked to assess different sets of videos of horses being loaded for road transport using both their own descriptors and a list of descriptors. Our results showed that giving first the possibility to observer to use their own descriptor is useful to the development and implementation of a fixed list with terms that perfectly fit with the specific conditions we want to evaluate. This would allow the observers to better understand and quantify each given descriptor and therefore obtain a good QBA accuracy level. Furthermore, a specific descriptors list was developed to be used in pre-transport loading situations.

**Abstract:**

To identify feasible indicators to evaluate animals’ emotional states as a parameter to assess animal welfare, the present study aimed at investigating the accuracy of free choice profiling (FCP) and fixed list (FL) approach of Qualitative Behaviour Assessment (QBA) in horses during the loading phase of transport. A total of 13 stakeholders were trained to score 2 different sets of videos of mixed breed horses loaded for road transport, using both FCP and FL, in 2 sessions. Generalized Procustes Analysis (GPA) consensus profile explained a higher percentage of variation (80.8%) than the mean of 1000 randomized profiles (41.2 ± 1.6%; *p* = 0.001) for the FCP method, showing an excellent inter-observer agreement. GPA identified two main factors, explaining 65.1% and 3.7% of the total variation. Factor 1 ranging from ‘anxious/ to ‘calm/relaxed’, described the valence of the horses’ emotional states. Factor 2, ranging from ‘bright’ to ‘assessing/withdrawn’, described the arousal. As for FL, Principal Component Analysis (PCA) first and second components (PC1 and PC2, respectively), explaining on average 59.8% and 12.6% of the data variability, had significant agreement between observers. PC1 ranges from relaxed/confident to anxious/frightened, while PC2 from alert/inquisitive to calm. Our study highlighted the need for the use of descriptors specifically selected, throughout a prior FCP process for the situation we want to evaluate to get a good QBA accuracy level.

## 1. Introduction

Horses are among the most transported animals in Europe [1]. Transportation is considered one of the most stressful event in an animal’s life and, therefore, it represents a major welfare concern in livestock [2]. In particular, the loading phase is one of the most stressful stages of animal transport [3,4] both in meat and sport horses. Meat horses have to deal with a series of new experiences, such as being handled by humans, being mixed with unfamiliar animals and entering a novel environment [5]. Moreover, several studies have shown that even sport horses, which are usually more used to transport, are particularly sensitive to the loading phase, showing strong negative reactions [6]. To evaluate the effect of transport procedures on equine stress, behavioral and physiological indicators can be used, such as cortisol level variations [7,8,9], glucose concentrations [10], circulating T3, T4 and fT4 levels [11,12], heart rate [11,13], core temperature [10], neutrophil: lymphocyte ratio [10], packed cell volume [13], cecal microbiota [14], evaluation of frequency and/or durations of stress-related behaviors [15,16,17]. The various physiological measurements were actually tested during transport of some duration rather than just the loading phase, which may cause transitory or minimal levels of stress to which these tests may not be sufficiently sensitive. Over the past few years, due to changes in societal belief and values and improved scientific understanding of animals, researchers are asked for identifying animal-based indicators to assess animals’ emotional states and their association with animal welfare assessment [18,19,20,21]. Qualitative behavior assessment (QBA) has been recently used as a tool to evaluate the affective state of animals during transport [22,23,24,25] and distinguish between groups of animals accustomed and naïve to transport [10,26]. QBA method requires scoring individuals’ or group of animals’ behaviors using different range of both positive and negative qualitative terms (i.e., apathetic, aggressive, content) on a Visual Analogue Scale [27,28,29]. This allows for identifying differences in animals’ emotional states expressed in valence, which expresses the mood (positive or negative) and arousal, which defines the level of excitement (high or low) [30,31]. QBA has been utilized within several animal species (including cattle [10,27,32,33,34], sheep [35,36], pigs [37,38,39], goats [21,40], horses [41,42], and donkeys [43,44]), in different farming systems, and has been found to correlate with other welfare indicators [21,32,34,38,43]. 

Evaluation of behavior using QBA can utilize two different approaches. In the Free Choice Profiling (FCP) methodology, observers are required to generate their own descriptors, based on observation of single animals or groups in a test situation [29,45]. To do so, observers watch video clips of animals in the test settings; at the end of each clip, they are required to write down adjectives they perceive adequately described how animals behaved in the clip. In this manner, each observer generates a personal list of descriptors, which is then used to quantitatively score the intensity of perceived animal’s expressions, watching the same video material a second time [29,45]. Subsequently, a multivariate statistical technique (Generalized Procrustes Analysis, GPA) is used to calculate the agreement among observers and to identify the commonly perceived dimensions of behavioral expression underlying the observers’ separate assessments [29]. In the fixed list approach, a pre-determined list of QBA terms is given to observers to assess animals’ behavior. To create the list, researchers select a number of qualitative terms from the relevant scientific literature on emotions, behavior, personality and temperament of the species in analysis. Then, experts, caretakers and other stakeholders are required to evaluate the appropriateness, relevance, and ease of understanding of selected terms, by applying the list to score several video clips of animals in the test settings. Finally, a definitive list is generated, including the terms evaluated as appropriate, relevant, and comprehensible [46,47,48]. Data collected with fixed list QBA are generally analyzed using Principal Component Analysis (PCA) to extrapolate the main dimensions of the animals’ emotional states [34,43].

FCP permits observers to integrate and judge the animals’ expressions, without being biased by provided terms [45]. Several research demonstrated good agreement among observers and a high level of accuracy in repeated assessment [27,42,49]. However, for on-farm monitoring of welfare or integration with assessment protocols, the use of FCP is not practical [48]. When a more standardized assessment is required, the use of a fixed-list is more feasible than FCP [28,46]. Advantages of using a fixed list for QBA evaluation in on-farm assessment include easier application and a straightforward interpretation of results by experienced assessors and experts [34]. Fixed QBA lists are included in several on-farm welfare assessment protocols, as a measure for both positive and negative emotional states [50,51,52,53,54,55]. A fixed list of QBA descriptors has also been developed and included in the AWIN welfare assessment protocol for horses [56]. 

To the authors’ knowledge, QBA has never been tested in horses during transport procedures; therefore, a specific list of descriptors to be used in horses subjected to transport has never been developed. To identify valid indicators to assess animal emotional states and to assess if they may be feasible to evaluate welfare of horses during the loading phase of transport, the present study aimed at assessing the accuracy of QBA by using the FCP and the fixed list approaches.

## 2. Materials and Methods

### 2.1. Animals and Video-Clips

A total of 40 videos of horses during the loading phase of transport were selected from a pool of videos collected during previous studies; videos have been recorded using a digital video camera (Canon Legria HFR88, Canon Inc., Tokyo, Japan), controlled by the experimenter. The entire loading procedure was video-recorded for each horse. Length of videos ranged from 39 to 110 s (mean = 50 s).

Videos were divided as follows:

- A total of 28 videos of Spanish Breton meat horses, of both sexes (M = 14; F = 14), aged 15 ± 2.79 months (min = 12 month; max = 24 months), from a meat horse farm located in North Eastern Italy. Horses were transported to the slaughterhouse, two to four horses at a time, using the same truck, according to the farm’s ordinary routine. Transport took place in the afternoon (~4:00 p.m.) on different days from April to October 2018. The farm manager performed the usual loading procedures, which involved minimal handling of horses: moving fences to let horses enter the loading lane, inciting horses from behind using voice and moving a stick only when they refused to move. More information regarding this group of horses can be found in Dai and colleagues’ study [15];
- A total of 12 videos of Arabian horses, of both sexes (M = 5, F = 7), aged 2.66 ± 1.77 years (min = 1 year; max = 7 years), from an equestrian center located in Northern Italy. The youngest horse was previously transported only once, all the remaining horses were previously transported to different competitions. All the horses were trained by the same person, but no specific training to load was in place. Horses were transported to show competitions, three to seven horses at a time, using the same truck, according to the farm’s ordinary routine. Transport took place in the afternoon (~2:00 p.m.) on different days from August to November 2019. The farm manager or a groom performed the usual loading procedures, which involved leading horses using headcollar and lead rope, inciting horses from behind using voice, moving a whip, and touching the back of the horse with a whip only when they refused to move.

### 2.2. Assessors and Observation Session

A total of 13 members of a horse welfare protection organization were recruited as assessors (11 females and 2 males). All assessors had previous extensive experience with horses, while none had previous experience with QBA. All assessors were English native speakers.

Three tutors were involved in the study, to educate assessors regarding the use of QBA and guide the observation sessions. Tutors were female veterinarians, expert in applied ethology and welfare evaluation of equids, with previous experience in using QBA in horses both for research and welfare assessment purposes.

Observations were organized in three sessions: an introductory section, a first video assessment session using FCP and a second video assessment session using a fixed list of terms. All the sessions were held live on-line, using Microsoft Teams (© Microsoft 2022) due to global pandemic. 

#### 2.2.1. Introductory Session

Before starting the assessment of the video clips, an introductory session, lasting approximately 1 h, was organized. In this first session the assessors were introduced to the concept of QBA and to the operative procedures of the study. Tutors explained the QBA background and how it has been used for on-farm welfare assessment in different species.

#### 2.2.2. Free-Choice Profile Session

In the first observation session, FCP was chosen, to ensure that assessors were not biased by predefined terms. Tutors played the set of 15 videos, including both show and meat horses during loading. After watching each video, assessors were asked to use descriptors to illustrate expressive styles of horse behavior in that specific video. Assessor could ask to re-watch each video clip twice. After watching all the videos, each assessors compiled their own unique list of descriptors. Assessors were then provided with a personal link for data collection sheet (see Section 2.2.5 for more information), containing their own terms to be scored on a Visual Analogue Scale (125 mm continuous scale). The same set of videos was played, and assessors were asked to score horse behavior using their own list of descriptors. Tutors explained how to use the scale: the left represented the ‘minimum’ and the right the ‘maximum’ point of the scale; a very left position on the scale or a zero indicated that the expressed quality of the specific descriptor was “entirely absent”, whereas the ‘maximum’ at 125 mm stood for “the expressed quality of the specific descriptor was constantly obvious during the observation”. 

#### 2.2.3. Fixed List Creation

Following the FCP session, tutors collected the lists generated by each assessor and collapsed them in a single list containing all the identified descriptors. Starting from this list, tutors moderated a discussion regarding the meaning of each descriptor. Assessors were asked to define each term and they were able to discuss until they reached a group consensus in the interpretation of each term. Following this session, a fixed list of 21 descriptors with definition was generated (Table 1). 

#### 2.2.4. Fixed List Session

The generated fixed list was then used in the last session to score the set of 25 videos of horses kept for meat production during loading procedure. Assessors received a link for data collection sheet (see Section 2.2.5 for more information), containing the fixed list of terms to be scored on a 125 mm Visual Analogue Scale.

#### 2.2.5. Data Collection

Online proprietary software (Qualtrics, Provo, Utah, UT, USA) was used to build data collection sheets and distribute them. As for FCP session, one data collection sheet for each assessor was built. The sheet contained the personal list of generated terms, each followed by a Visual Analogue Scale (1 to 125 mm long). Each participant received via e-mail a personal link to access to their sheet. Following the Fixed List creation, one data collection sheet was created, containing the fixed list of descriptors, each followed by a Visual Analogue Scale (1 to 125 mm long). The link to access this sheet was distributed to all assessors.

### 2.3. Statistical Analysis

The inter-observer agreement in the FCP was investigated using Generalized Procrustes Analysis (GPA), a multivariate statistical technique that does not rely on fixed variables. GPA transforms individual observer scoring patterns into multidimensional configurations, which are made comparable with each other through sequence of rototranslations and rescalings, determines the “mean” of these configurations, named the “best fit” or “consensus profile”. This calculation is essentially a process of pattern recognition and takes place independently of the meaning of the terminologies used by assessors. How well individual assessor scores fit the consensus profile (i.e., the degree of agreement) is quantified by the Procrustes Statistic and visually represented by an ‘observer plot’ [29].

To analyze inter-observer reliability for each descriptor of the generated QBA fixed list, Kendall Correlation Coefficient W was calculated on the raw descriptor scores. Kendall W values can vary from 0 (no agreement at all) to 1 (complete agreement). The following threshold [57] were used to interpret Kendall’s W:

- 0–0.2 poor agreement;
- 0.2–0.4 fair agreement;
- 0.4–0.6 moderate;
- 0.6–0.8 good;
- 0.8–1 very good.

Bonferroni procedure was used to adjust *p*-values. 

For the subsequent analyses, those descriptors with Kendall’s W < 0.20 (poor agreement between raters) were excluded, namely “Aggressive”, “Excited”, and “Withdrawn”. A Principal Component Analysis (PCA, correlation matrix) was performed on the remaining descriptors (*n* = 18) for each assessor separately. Analyses were conducted using R software (version 3.6.1) and “FactoMineR” and “irr” packages.

## 3. Results & Discussion

### 3.1. Free Choice Profile

**Figure 1:**
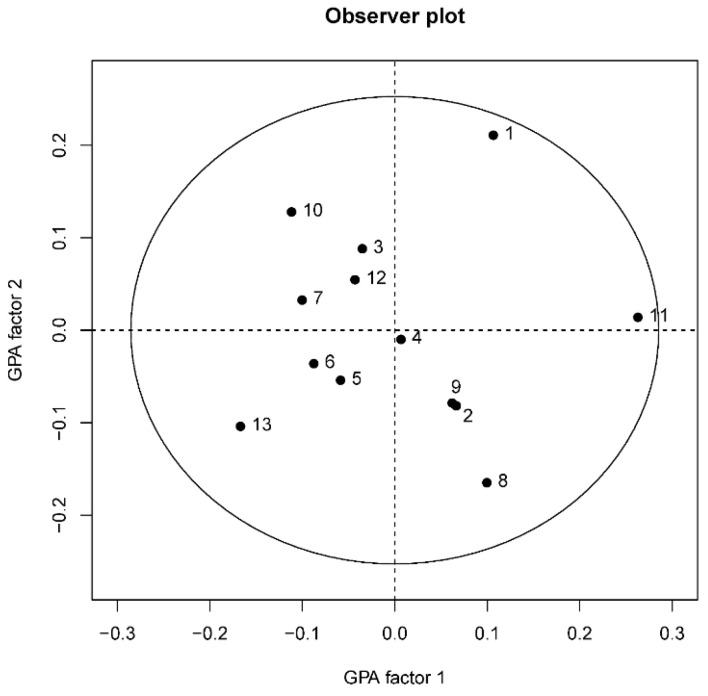
Observer plot shows the consensus among the 13 QBA assessors using the FCP methodology, the circle represents within the 95% confidence region.

The FCP methodology allowed the observers to generate their own unique set of terms to describe behavioral expression and then use it to score the behavior of fifteen mixed horses at the loading phase of transport. The Procrustes Statistic of the GPA consensus profile explained a significantly higher percentage of variation (80.8%) than the mean of 1000 randomized profiles (41.2 ± 1.6%; *p* = 0.001), indicating the consensus to be a significant feature of the dataset rather than an artefact of the Procrustean calculation procedures. The observer plot (Figure 1) reflects the consensus among the 13 observers, as all of them fall within the 95% confidence region. Thus, showing an excellent inter-observer agreement of the FCP method, in line with what has been already shown in other species [27,42,49,58,59], and generally supporting the reliability of the QBA methodology. 

Two main factors of the consensus profile were identified, explaining 65.1% and 3.7% of the total variation between animals, respectively, in accordance with the vast majority of the studies that applied the FCP to different species [27,28,39,41,42,49,58] that found two main dimensions. To provide an overview of highly correlated terms (correlation ≥ 0.60) for all assessors, Table 1 lists the most used descriptors (their frequencies over the 13 observers) with the highest positive and negative correlation to factors 1 and 2 of the consensus profile. On the basis of these results, GPA factor 1 (GPA1) was qualitatively labelled as ranging from ‘anxious/tense’ (positive correlation) to ‘calm/relaxed’ (negative correlation), and GPA factor 2 (GPA2) as ranging from ‘bright’ at the high end of the axes to ‘assessing/withdrawn’ at the low end. GPA1 describes the valence of the horses’ emotional states, while GPA2 tried to explain the arousal. Most of the terms used by our observers were then labelled as belonging to GPA1 and distinguish between horses that seemed tense/anxious (chosen by 11 and 10 observers, respectively) or even scared (chosen by 6 observers) during the loading phase and horses that seemed to be calm/relaxed (11 observers each) or even willing (reported by 3 observers). Calm vs. activation/agitation is one of the most common GPA factors also in other FCP studies even when used to evaluate other species assessed under different conditions [22,39,58,60,61,62]. On the other hand, very few participants reported descriptors that were then labelled in GPA2: only one observer rated a horse as “bright” and the adjectives “assessing”, “depressed” and “withdrawn” were used once each. In the willingness of QBA to assess how the animal is behaving and therefore catch its affective state [21,41], when applied to loading situations the most used terms to describe the emotional valence that we collected seem to embed themselves an arousal variation. More to the point, it could be because an animal habituated and confident to be loaded react less compared to one that feels uncomfortable or even scared. This is particularly true for horses, that are well-known for their typical flight-wired reactions [63]. Horses afraid by the confinement that the trailer imposes indeed exhibit behaviors, such as rearing, pulling back, head tossing, pawing, and turning sideways [6]. Therefore, for example, it would be quite difficult to find descriptors with low valence and low arousal, such as “apathetic” or “annoyed”, when the animal is not comfortable with transport, especially during this phase. Or vice versa descriptors with high valence and high arousal, such as “happy” or “look for contact”. Apathetic, annoyed, happy, and look for contact are all descriptors that actually belong to the AWIN QBA fixed list [56]. If we had designed our study using that fixed list first, it is likely that our observers would have struggled to use these terms that do not fit well with the possible loading reactions of horses. This has been also hypothesized by Napolitano and colleagues in their study on dairy buffalos, where if terms were imposed on observers through pre-determined scoring lists, agreement would not be as high as found in that study, or would be high for some terms but not others [58]. 

On the other hand, it is well recognized that the use of long and complex lists may be difficult for different assessors to be fully understood and implemented [28,48], while a more concise list may also be easier and faster to complete.

**Figure 2:**
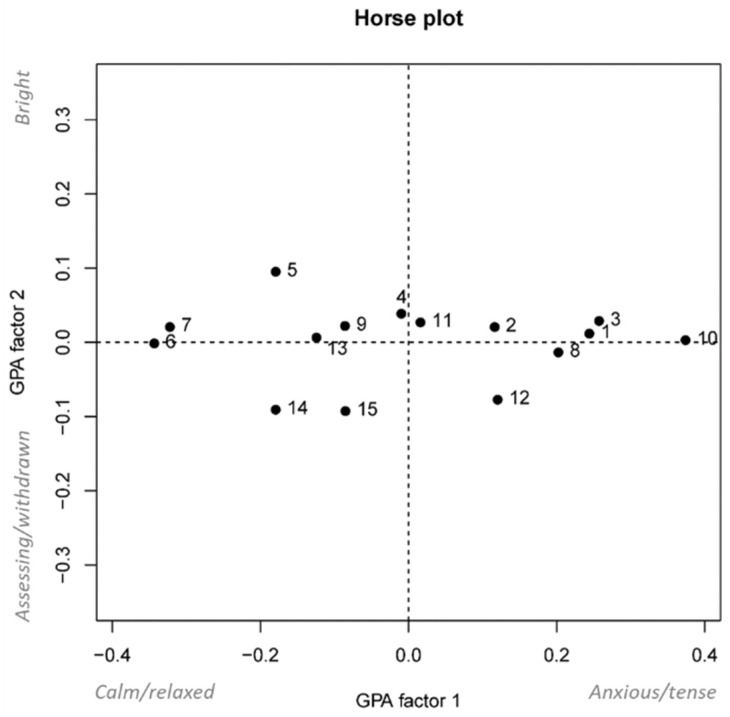
Horse plot shows the individual horses positioned on the two main GPA factors.

Moreover, analyzing the numerosity of the GPA1 descriptors, there is a quite higher variability and numerosity in the list of terms with positive correlation of GPA1 (13 positive correlated descriptors vs. 5 negative correlated descriptors). Different raters found many different shades in the demeanor of those horses that were not comfortable at being loaded, while “calm”, “relaxed” and “confident” were the mainly used terms to describe cooperative or not stressed horses. This could be because our observers tended to focus more on problematic behaviors and horses compared to quiet situations. Despite that, we found a clear semantic correlation between the terms used to describe each horse. Therefore, in addition to the high level of inter-observer agreement, QBA may be a tool worth considering assessing emotional states variation in valence and arousal. Or, at least, it is easier to reach a consensus on it, even in the event that all the observers were collectively wrong as hypothesized by Wemelsfelder and colleagues [29]. Figure 2 shows the ‘horse plot’ of the QBA in which individual horses (showed in the first 15 score videos) are positioned on the two main factors of the GPA consensus profile. These positions and the variation between them can be semantically interpreted with the qualitative labels discussed above. Once more, it seems evident that one GPA factor, GPA dimension 1, is more “influential” and variable than the other, indicating a different observer ability to catch variation in the arousal not linked to emotional valence variability. 

### 3.2. Fixed Term List

Although FCP methodology removes possible bias due to provided terms allowing each observers to use its own terms, it is well recognized that fixed lists are more practical, feasible, and suitable than FCP for on-farm QBA’s implementation [28,48], when a standardized way of assessment is needed for feasibility reasons. Therefore, starting from FCP results, we built a Fixed List, reported in Table 2 and then tested inter-observer reliability of each term after the observers used them to describe a second group of 25 horses. It is important that before they use the list, all the observers reach consensus on the meaning of each term, in order to remove any linguistic barriers or misunderstanding [59] and then eliminate those terms that fail to reach good agreement.

Kendall’s W results are reported in Table 3. For the subsequent analyses, those descriptors with Kendall’s W < 0.20 (poor agreement between raters) were excluded, namely “Aggressive”, “Excited”, and “Withdrawn”. 

**Figure 3:**
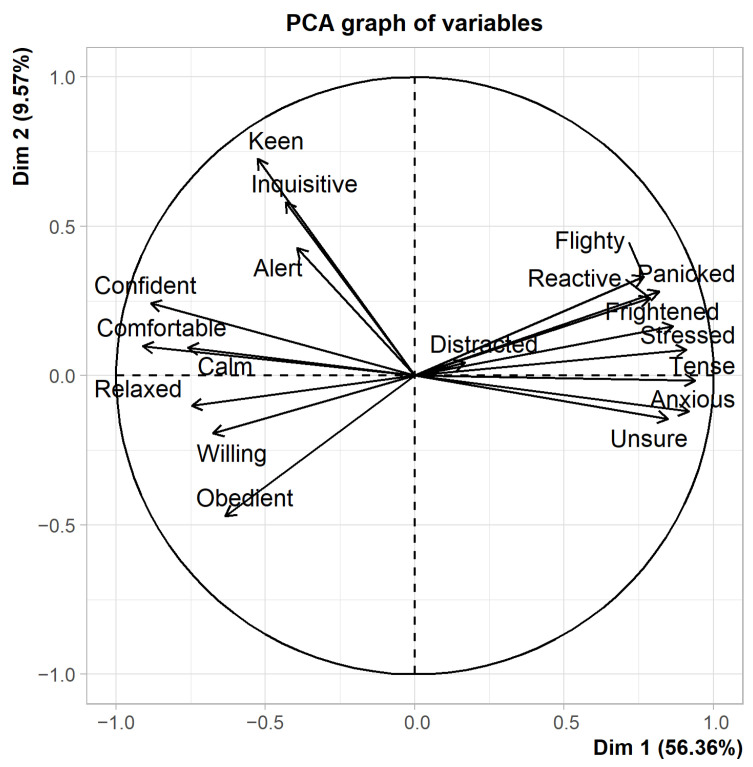
Loading plot of QBA fixed list descriptors along the first two PCA components for Assessor 2.

A Principal Component Analysis (PCA, correlation matrix) was performed on the remaining descriptors (*n* = 18) for each assessor separately. Then, we decided not to consider descriptors that were only mentioned once in each principal component (PC). Figure 3 provides an example of a PCA graph performed on a single assessor, with the first dimension on the x-axis and the second one on the y-axis. It is immediately evident that PC1 is the one that varies more.

Results showed that first and second PCs had both significant agreement between raters in terms of loadings and scores, and these two PCs explained on average 59.8% and 12.6% of the data variability, respectively (Table 4). The agreement on the third PC (which explained a lower percentage of variance) was poor and non-significant. PC1 ranges from relaxed/confident to anxious/frightened, while PC2 from alert/inquisitive to calm. Thus, both in FCP and in FL method GPA dimension 1 generally demonstrates a valence of mood with “relaxed/confident” vs. “anxious/tense/frightened”, in accordance with what has already been described by Clarke and colleagues [60]. In addition, even with the FL the percentage of explained variance is higher for PC1, linked to valence, compared to PC2, that described the arousal. This again confirms that in this specific situation, the loading phase of transport, with this specific species, horses, observers were not so able to distinguish arousal variability independently of the pleasantness, but they tended to use terms that embedded an arousal description. 

The lower agreement, compared to the one reached in the FCP phase, could be because observers were required to use fixed terms, as assumed also by Napolitano and colleagues [58]. This semantical consensus and the inter-observer agreement once again makes QBA a tool worth considering when assessing if a horse is calm and confident during loading or if otherwise it is worried and afraid.

## 4. Conclusions

As societal beliefs and values are changing and our knowledge on animal sentience is growing, a positive animal welfare approach is increasingly needed to meet these new needs. Therefore, in order to identify new feasible indicators to evaluate animals’ positive and negative emotional states as a parameter to assess animal welfare, the aim of our study was assessing the accuracy of both FCP and FL QBA approaches in horses during the sensitive phase of pre-road transport loading. It is well-known that the FCP methodology frees the observers from bias and misunderstandings and allows him/her to describe how the animal is behaving with the words he/she most prefers. On the other hand, the use of fixed lists is more practical and, thus, why it is the first-choice method in on-farm situations. Our results showed the importance of developing both these methodologies under the specific conditions we would use it to get a list of terms that fit. More specifically, both in FCP and in FL most of the terms that our observers used were labelled in the “valence dimension”, while they struggled to catch arousal variation. This is most likely related to how a horse tends to deal with the loading phase: its body activation tends to increase the more it feels uncomfortable, vice versa its movements are minimized if it is calm and relaxed at the idea of being loaded. Furthermore, a more concise list may be easier and faster to complete in on farm conditions. Thus, our findings highlighted the need for the use of descriptors specifically selected, throughout a prior FCP process, for the situation we want to evaluate to get a good QBA accuracy level.

## Figures and Tables

**Table 1:** The descriptors most used by the assessors (frequencies over the 13 observers) with the highest positive and negative correlation to factors 1 and 2 of the consensus profile.

GPA Factor	Positive Correlation (≥0.60)		Negative Correlation (≥0.60)	
	Descriptor	Freq	Descriptor	Freq
GPA factor 1	Tense	11	Calm	11
	Anxious	10	Relaxed	11
	Unsure	9	Confident	10
	Nervous	9	Curious	3
	Stressed	8	Willing	3
	Scared	6		
	Agitated	6		
	Worried	4		
	Wary	4		
	Resistant	4		
	Frightened	4		
	Alert	4		
	Flighty	3		
GPA factor 2	Bright	1	Assessing	1
			Depressed	1
			Withdrawn	1

**Table 2:** Fixed term list developed by Free Choice Profiling mainly used terms. For each term, observers discussed and agreed on a brief description of the meaning.

Descriptor	Definition
Aggressive	Displaying hostile behaviors
Alert	Heightened interest, fully aware and attentive
Anxious	Nervous, unsure of its current situation; greatly worried
Calm	Quiet; not disturbed, agitated or excited
Comfortable	Content with situation and environment, moving freely
Confident	Not hesitant, showing certainty; assured
Distracted	Not paying attention, looking around or listening to something else
Excited	High energy state due to feeling anticipation or nervousness
Flighty	Naturally instinctive reactions to environment; highly strung
Frightened	Fearful or alarmed
Inquisitive	Curious; eager to learn; enquiring
Keen	Eager or enthusiastic, motivated
Obedient	Carrying out directions without hesitation or question; cooperative
Panicked	Fear from within the body, overwhelming feeling of terror or anxiety
Reactive	Over responsive to stimulus
Relaxed	Appears free of tension or anxiety; at ease
Stressed	Feeling mental, emotional, or physical strain or tension
Tense	Visibly uneasy at the situation, rigid posture, ready to react
Unsure	Hesitant response, not certain or confident
Willing	Wanting to respond to what is being asked
Withdrawn	Mentally or emotionally detached; shutdown

**Table 3:** Inter-rater agreement analysis (Kendall’s W and adjust *p*-values with Bonferroni procedure) and agreement interpretation.

Descriptor	Kendall’s W	$\chi^{2}\;\mathrm{Statistic}\;$ (24 df)	Adjusted *p*-Value	Agreement
Aggressive	0.0834	26.0	1	poor
Alert	0.3295	102.8	<0.0001	fair
Anxious	0.6286	196.1	<0.0001	good
Calm	0.6463	201.6	<0.0001	good
Comfortable	0.5219	162.8	<0.0001	moderate
Confident	0.6117	190.9	<0.0001	good
Distracted	0.2374	74.1	<0.0001	fair
Excited	0.1752	54.7	0.0073	poor
Flighty	0.4284	133.7	<0.0001	moderate
Frightened	0.6689	208.7	<0.0001	good
Inquisitive	0.4121	128.6	<0.0001	moderate
Keen	0.4300	134.1	<0.0001	moderate
Obedient	0.4789	149.4	<0.0001	moderate
Panicked	0.6084	189.8	<0.0001	good
Reactive	0.4679	146.0	<0.0001	moderate
Relaxed	0.5252	163.9	<0.0001	moderate
Stressed	0.5704	178.0	<0.0001	moderate
Tense	0.6193	193.2	<0.0001	good
Unsure	0.5558	173.4	<0.0001	moderate
Willing	0.4063	126.8	<0.0001	moderate
Withdrawn	0.1726	53.8	0.0094	poor

**Table 4:** Summary results of PCA of the descriptors. Lists of correlated descriptors include only those with a loading ≥ 0.2 in absolute value.

	Inter-Rater Agreement, Kendall’s W (*p*-Value)		Percentage of Explained Variance, Mean ± St. Dev. (Range) across Raters	Correlated Descriptors (Number of Raters)	
	On Scores	On Loadings		Positive Corr.	Negative Corr.
PC1	0.771 (<0.0001)	0.914 (<0.0001)	59.8 ± 10.2 (41.1–77.1)	Calm (13) Confident (13) Comfortable (12) Relaxed (12) Willing (11) Keen (9) Obedient (7) Inquisitive (5)	Anxious (13) Frightened (13) Panicked (13) Stressed (13) Tense (13) Unsure (13) Flighty (8) Reactive (8) Alert (4)
PC2	0.201 (<0.0001)	0.225 (<0.0001)	12.6 ± 5.3 (6.4–24.1)	Alert (10) Inquisitive (9) Keen (8) Distracted (7) Flighty (7) Obedient (6) Reactive (6) Willing (4) Confident (3) Panicked (3) Comfortable (2)	Calm (2) Flighty (2) Reactive (2)
PC3	0.066 (0.659)	0.121 (0.063)	7.5 ± 2.1 (4.7–11.5)	Alert (7) Distracted (6) Inquisitive (5) Obedient (4) Comfortable (3) Flighty (3) Keen (3) Panicked (3) Reactive (3) Willing (3)	Distracted (4) Reactive (4) Flighty (3) Inquisitive (3) Unsure (3) Willing (3) Comfortable (2) Stressed (2) Tense (2)

## Data Availability

The data that support the findings of this study are available on request from the corresponding author E.D.C.

## References

[1] World Horse Welfare (2015). Eurogroup for Animals Removing the Blinkers: The Health and Welfare of European Equidae in 2015. https://www.eurogroupforanimals.org/files/eurogroupforanimals/2020-02/EU-Equine-Report-Removing-the-Blinkers_0.pdf.

[2] Broom D.M. (2005). The effects of land transport on animal welfare. OIE Rev. Sci. Tech..

[3] Trunkfield H.R., Broom D.M. (1990). The welfare of calves during handling and transport. Appl. Anim. Behav. Sci..

[4] Tateo A., Padalino B., Boccaccio M., Maggiolino A., Centoducati P. (2012). Transport stress in horses: Effects of two different distances. J. Vet. Behav..

[5] Šímová V., Večerek V., Passantino A., Voslářová E. (2016). Pre-transport factors affecting the welfare of cattle during road transport for slaughter—A review. Acta Vet. Brno.

[6] Ferguson D.L., Rosales-Ruiz J. (2001). Loading the problem loader: The effects of target training and shaping on trailer-loading behavior of horses. J. Appl. Behav. Anal..

[7] White A., Reyes A., Godoy A., Martínez R. (1991). Effects of transport and racing on ionic changes in thoroughbred race horses. Comp. Biochem. Physiol. Part A Physiol..

[8] Leadon D.P. (1995). Transport stress and the equine athlete. Equine Vet. Educ..

[9] Ferlazzo A., Fazio E., Murania C., Piccione G. Physiological Responses of Stallion to Transport Stress. Proceedings of the 3rd International Congress of the International Society for Applied Ethology.

[10] Stockman C.A., Collins T., Barnes A.L., Miller D., Wickham S.L., Beatty D.T., Blache D., Wemelsfelder F., Fleming P.A. (2011). Qualitative behavioural assessment and quantitative physiological measurement of cattle naïve and habituated to road transport. Anim. Prod. Sci..

[11] Fazio E., Medica P., Cravana C., Giacoppo E., Ferlazzo A. (2009). Physiological variables of horses after road transport. Animal.

[12] Aronica V., Medica P., Cusumano F., Fazio E. Effect of Transport Stress and Influence of Distance, Age and Breed on the Thyroid Function of Horses. Proceedings of the SISVET Annual Meeting.

[13] Padalino B., Maggiolino A., Boccaccio M., Tateo A. (2012). Effects of different positions during transport on physiological and behavioral changes of horses. J. Vet. Behav..

[14] Perry E., Cross T.W.L., Francis J.M., Holscher H.D., Clark S.D., Swanson K.S. (2018). Effect of Road Transport on the Equine Cecal Microbiota. J. Equine Vet. Sci..

[15] Dai F., Costa A.D., Bonfanti L., Caucci C., Di Martino G., Lucarelli R., Padalino B., Minero M. (2019). Positive reinforcement-based training for self-loading of meat horses reduces loading time and stress-related behavior. Front. Vet. Sci..

[16] Padalino B., Raidal S.L., Knight P., Celi P., Jeffcott L., Muscatello G. (2018). Behaviour during transportation predicts stress response and lower airway contamination in horses. PLoS ONE.

[17] WARAN N.K. (1993). The behaviour of horses during and after transport by road. Equine Vet. Educ..

[18] Lassen J., Sandøe P., Forkman B. (2006). Happy pigs are dirty!—Conflicting perspectives on animal welfare. Livest. Sci..

[19] Robbins J., Franks B., von Keyserlingk M.A.G. (2018). ‘More than a feeling’: An empirical investigation of hedonistic accounts of animal welfare. PLoS ONE.

[20] Vigors B. (2019). Citizens’ and Farmers’ Framing of ‘Positive Animal Welfare’ and the Implications for Framing Positive Welfare in Communication. Animals.

[21] Battini M., Barbieri S., Vieira A., Can E., Stilwell G., Mattiello S. (2018). The use of qualitative behaviour assessment for the on-farm welfare assessment of dairy goats. Animals.

[22] Wickham S.L., Collins T., Barnes A.L., Miller D.W., Beatty D.T., Stockman C.A., Blache D., Wemelsfelder F., Fleming P.A. (2015). Validating the Use of Qualitative Behavioral Assessment as a Measure of the Welfare of Sheep During Transport. J. Appl. Anim. Welf. Sci..

[23] Wickham S. (2011). Qualitative Behavioural Assessment of Sheep during Transport. Ph.D. Thesis.

[24] Stockman C.A., Collins T., Barnes A.L., Miller D., Wickham S.L., Beatty D.T., Blache D., Wemelsfelder F., Fleming P.A. (2013). Flooring and driving conditions during road transport influence the behavioural expression of cattle. Appl. Anim. Behav. Sci..

[25] Collins T., Stockman C.A., Barnes A.L., Miller D.W., Wickham S.L., Fleming P.A. (2018). Qualitative behavioural assessment as a method to identify potential stressors during commercial sheep transport. Animals.

[26] Wickham S.L., Collins T., Barnes A.L., Miller D.W., Beatty D.T., Stockman C., Blache D., Wemelsfelder F., Fleming P.A. (2012). Qualitative behavioral assessment of transport-naïve and transport-habituated sheep. J. Anim. Sci..

[27] Rousing T., Wemelsfelder F. (2006). Qualitative assessment of social behaviour of dairy cows housed in loose housing systems. Appl. Anim. Behav. Sci..

[28] Wemelsfelder F., Millard F., de Rosa G., Napolitano F. (2009). Qualitative behaviour assessment. In: Assessment of animal welfare measures for layers and broilers. Welf. Qual. Rep..

[29] Wemelsfelder F., Hunter E.A., Mendl M.T., Lawrence A.B. (2000). The spontaneous qualitative assessment of behavioural expressions in pigs: First explorations of a novel methodology for integrative animal welfare measurement. Appl. Anim. Behav. Sci..

[30] Fleming P.A., Clarke T.A., Wickham S.L., Stockman C.A., Barnes A.L., Collins T.A., Miller D.W. (2016). The contribution of qualitative behavioural assessment to appraisal of livestock welfare. Anim. Prod. Sci..

[31] Mendl M., Burman O.H.P., Paul E.S. (2010). An integrative and functional framework for the study of animal emotion and mood. Proc. R. Soc. B Biol. Sci..

[32] Brscic M., Wemelsfelder F., Tessitore E., Gottardo F., Cozzi G., van Reenen C.G. (2009). Welfare assessment: Correlations and integration between a Qualitative Behavioural Assessment and a clinical/ health protocol applied in veal calves farms. Ital. J. Anim. Sci..

[33] Ceballos M.C., Góis K.C.R., Sant’Anna A.C., Wemelsfelder F., Paranhos da Costa M. (2021). Reliability of qualitative behavior assessment (QBA) versus methods with predefined behavioral categories to evaluate maternal protective behavior in dairy cows. Appl. Anim. Behav. Sci..

[34] Brscic M., Otten N.D., Contiero B., Kirchner M.K. (2019). Investigation of a standardized qualitative behaviour assessment and exploration of potential influencing factors on the emotional state of dairy calves. Animals.

[35] Phythian C., Michalopoulou E., Duncan J., Wemelsfelder F. (2013). Inter-Observer Reliability of Qualitative Behavioural Assessments of Sheep. Appl. Anim. Behav. Sci..

[36] Phythian C.J., Michalopoulou E., Cripps P.J., Duncan J.S., Wemelsfelder F. (2016). On-farm qualitative behaviour assessment in sheep: Repeated measurements across time, and association with physical indicators of flock health and welfare. Appl. Anim. Behav. Sci..

[37] Wemelsfelder F. (2012). Assessing pig body language: Agreement and consistency between pig farmers, veterinarians, and animal activists. J. Anim. Sci..

[38] Duijvesteijn N., Benard M., Reimert I., Camerlink I. (2014). Same Pig, Different Conclusions: Stakeholders Differ in Qualitative Behaviour Assessment. J. Agric. Environ. Ethics.

[39] Rutherford K.M.D., Donald R.D., Lawrence A.B., Wemelsfelder F. (2012). Qualitative Behavioural Assessment of emotionality in pigs. Appl. Anim. Behav. Sci..

[40] Grosso L., Battini M., Wemelsfelder F., Barbieri S., Minero M., Dalla Costa E., Mattiello S. (2016). On-Farm Qualitative Behaviour Assessment of Dairy Goats in Different Housing Conditions. Appl. Anim. Behav. Sci..

[41] Minero M., Tosi M., Canali E., Wemelsfelder F. (2009). Quantitative and Qualitative Assessment of the Response of Foals to the Presence of an Unfamiliar Human. Appl. Anim. Behav. Sci..

[42] Napolitano F., De Rosa G., Braghieri A., Grasso F., Bordi A., Wemelsfelder F. (2008). The Qualitative Assessment of Responsiveness to Environmental Challenge in Horses and Ponies. Appl. Anim. Behav. Sci..

[43] Minero M., Dalla Costa E., Dai F., Murray L.A.M., Canali E., Wemelsfelder F. (2016). Use of Qualitative Behaviour Assessment as an indicator of welfare in donkeys. Appl. Anim. Behav. Sci..

[44] Dai F., Dalla Costa E., Anne Murray L.M., Canali E., Minero M. (2016). Welfare Conditions of Donkeys in Europe: Initial Outcomes from On-Farm Assessment. Animals.

[45] Wemelsfelder F. (2007). How Animals Communicate Quality of Life: The Qualitative Assessment of Behaviour. Anim. Welf..

[46] Arena L., Wemelsfelder F., Messori S., Ferri N., Barnard S. (2019). Development of a fixed list of terms for the qualitative behavioural assessment of shelter dogs. PLoS ONE.

[47] Muri K., Stubsjøen S.M. (2017). Inter-observer reliability of Qualitative Behavioural Assessments (QBA) of housed sheep in Norway using fixed lists of descriptors. Anim. Welf..

[48] Wemelsfelder F., Mullan S. (2014). Applying ethological and health indicators to practical animal welfare assessment. Rev. Sci. Tech..

[49] Wemelsfelder F., Hunter T.E.A., Mendl M.T., Lawrence A.B. (2001). Assessing the “whole animal”: A free choice profiling approach. Anim. Behav..

[50] Welfare Quality Consortium Welfare Quality® Assessment Protocol for Cattle 2009. http://www.welfarequalitynetwork.net/media/1088/cattle_protocol_without_veal_calves.pdf.

[51] Welfare Quality Consortium Assessment Protocol for Poultry (Broilers, Laying Hens) (2009). Lelystad: Welfare Quality® Consortium. http://www.welfarequalitynetwork.net/media/1293/poultry-protocol-watermark-6-2-2020.pdf.

[52] Welfare Quality Consortium Welfare Quality® Assessment protocol for pigs 2009. http://www.welfarequalitynetwork.net/media/1018/pig_protocol.pdf.

[53] AWIN AWIN Welfare Assessment Protocol for Sheep 2015.

[54] AWIN AWIN Welfare Assessment Protocol for Goats 2015.

[55] AWIN AWIN Welfare Assessment Protocol for Donkeys 2015.

[56] AWIN AWIN Welfare Assessment Protocol for Horses 2015.

[57] Eliasson K., Palm P., Nyman T., Forsman M. (2017). Inter- and intra-observer reliability of risk assessment of repetitive work without an explicit method. Appl. Ergon..

[58] Napolitano F., De Rosa G., Grasso F., Wemelsfelder F. (2012). Qualitative behaviour assessment of dairy buffaloes (Bubalus bubalis). Appl. Anim. Behav. Sci..

[59] Walker J., Dale A., Waran N., Clarke N., Farnworth M., Wemelsfelder F. (2010). The assessment of emotional expression in dogs using a Free Choice. Anim. Welf..

[60] Clarke T., Pluske J.R., Fleming P.A. (2016). Are observer ratings influenced by prescription? A comparison of Free Choice Profiling and Fixed List methods of Qualitative Behavioural Assessment. Appl. Anim. Behav. Sci..

[61] Arena L., Wemelsfelder F., Messori S., Ferri N., Barnard S. (2017). Application of Free Choice Profiling to assess the emotional state of dogs housed in shelter environments. Appl. Anim. Behav. Sci..

[62] Fernandes J., Blache D., Maloney S.K., Martin G.B., Venus B., Walker F.R., Head B., Tilbrook A. (2019). Addressing Animal Welfare through Collaborative Stakeholder Networks. Agriculture.

[63] LeGuin E., Raber K., Tucker T.J. (2005). Man and Horse in Harmony. The Culture of the Horse: Status, Disciplin, and Identity in the Early Modern World.

